# Effects of narrow-wide row planting patterns on canopy photosynthetic characteristics, bending resistance and yield of soybean in maize‒soybean intercropping systems

**DOI:** 10.1038/s41598-024-59916-5

**Published:** 2024-04-23

**Authors:** Yan Gu, Haoyuan Zheng, Shuang Li, Wantong Wang, Zheyun Guan, Jizhu Li, Nan Mei, Wenhe Hu

**Affiliations:** 1 Jilin Agricultural University, Changchun, 131008 China; 2 Jilin Academy of Agricultural Sciences, Changchun, 130124 China

**Keywords:** Photosynthesis, Plant physiology

## Abstract

With the improvements in mechanization levels, it is difficult for the traditional intercropping planting patterns to meet the needs of mechanization. In the traditional maize‒soybean intercropping, maize has a shading effect on soybean, which leads to a decrease in soybean photosynthetic capacity and stem bend resistance, resulting in severe lodging, which greatly affects soybean yield. In this study, we investigated the effects of three intercropping ratios (four rows of maize and four rows of soybean; four rows of maize and six rows of soybean; six rows of maize and six rows of soybean) and two planting patterns (narrow-wide row planting pattern of 80–50 cm and uniform-ridges planting pattern of 65 cm) on soybean canopy photosynthesis, stem bending resistance, cellulose, hemicellulose, lignin and related enzyme activities. Compared with the uniform-ridge planting pattern, the narrow-wide row planting pattern significantly increased the LAI, PAR, light transmittance and compound yield by 6.06%, 2.49%, 5.68% and 5.95%, respectively. The stem bending resistance and cellulose, hemicellulose, lignin and PAL, TAL and CAD activities were also significantly increased. Compared with those under the uniform-ridge planting pattern, these values increased by 7.74%, 3.04%, 8.42%, 9.76%, 7.39%, 10.54% and 8.73% respectively. Under the three intercropping ratios, the stem bending resistance, cellulose, hemicellulose, lignin content and PAL, TAL, and CAD activities in the M4S6 treatment were significantly greater than those in the M4S4 and M6S6 treatments. Compared with the M4S4 treatment, these variables increased by 12.05%, 11.09%, 21.56%, 11.91%, 18.46%, 16.1%, and 16.84%, respectively, and compared with the M6S6 treatment, they increased by 2.06%, 2.53%, 2.78%, 2.98%, 8.81%, 4.59%, and 4.36%, respectively. The D-M4S6 treatment significantly improved the lodging resistance of soybean and weakened the negative impact of intercropping on soybean yield. Therefore, based on the planting pattern of narrow-wide row maize‒soybean intercropping planting pattern, four rows of maize and six rows of soybean were more effective at improving the lodging resistance of soybean in the semiarid region of western China.

## Introduction

Due to global warming, shortages of land resources, population growth and other unfavourable factors, agricultural production and sustainable agricultural development have become a difficult challenges^1^. In the current situation of a lack of land resources, agricultural intensification can effectively improve crop yield and alleviate resource shortages^2^. Intercropping is an important way to improve the utilization efficiency of resources^3^. It can achieve adequate distribution of light, temperature, water, gas and heat^4–6^, promote the rational utilization of land resources^7^, and is conducive to the sustainable development of agriculture^8^. Intercropping is also an effective strategy for increasing crop yields^9^.

Maize–soybean intercropping is an effective intercropping pattern^10^. Soybean is a nitrogen-fixing C3 crop, and maize is a nitrogen-consuming C4 crop; because of these differences, these crops can achieve complementary effects^11^. Under intercropping conditions, the maize‒soybean intercropping system improves light interception by intercropped species and accelerates their biomass production^12^. Light is one of the main factors for photosynthesis in crops. Crops convert light energy into chemical energy through photosynthesis for organic matter production^13^. The photosynthetically active radiation (PAR), leaf area index (LAI) and light transmittance are important indicators of crop photosynthesis^14^. The traditional intercropping mode effectively improves the light transmittance of maize and improves its photosynthetic capacity. However, due to the shading effect of maize on soybean, the photosynthesis in intercropped soybean is negative affected^15^.

Intercropped soybean is susceptible to shading stress from maize in the early, middle and late growth stages, resulting in soybean lodging^16^. Under the traditional intercropping, the light energy interception rate of soybean is significantly reduced and its photosynthetic capacity decreases^17^. Moreover, due to the shading effect of maize, soybean grows slender stems with reduced breaking resistance, resulting in soybean lodging^18^. Previous studies have shown that due to the shading effect of maize, soybean lodging occurs, leading to an unbalance in the proportion of dry matter accumulation, with greater dry matter distribution to vegetative organs and lower distribution to reproductive organs, resulting in a soybean yield reduction rate of approximately 54%^19^. Through the study of different maize‒soybean intercropping ratios of 2:2, 2:3, 2:4, and 2:5^20^, it was concluded that as the soybean intercropping ratio increased, the shading of soybean by maize gradually decreased. Under the 2:5 ratio, the photosynthetic capacity of soybean increased by 25.5% compared with that under the 2:2 ratio, and the nutrient content of soybean stems increased by 23.8%, which promoted the transfer of organic matter to the stem and improved its bending resistance. Another study showed that the shading effect of maize on soybean can be effectively alleviated by changing the row ratio. Compared with a maize‒soybean row ratio of 80:120 cm, the soybean competition ratio was increased by approximately 55% when the maize–soybean row ratio was adjusted to 20:160 cm, and the soybean yield was significantly improved. Additionally, the resource utilization efficiency of maize and soybean reached 3.26 mg/MJ^21^.

Western Jilin Province is a semiarid region with a long and continuous history of maize and soybean cultivation; however, currently, the soil productivity is reduced and the available nutrients are insufficient. Despite the continuous increase in mechanization, the traditional maize–soybean intercropping model has not been adapted to the mechanized process of modern crop production. Therefore, the main objectives of this study were to analyse which planting mode can improve the lodging resistance of soybean and to determine the effect of planting mode on the physiological indices of soybean lodging resistance by exploring wide-narrow row planting modes and different intercropping ratios. The lodging resistance and yield of soybean were comprehensively analysed to determine which planting mode can improve the resistance of soybean plants and weaken the negative effect of intercropping on soybean yield as much as possible.

## Results

### Leaf area index

**Figure 1:**
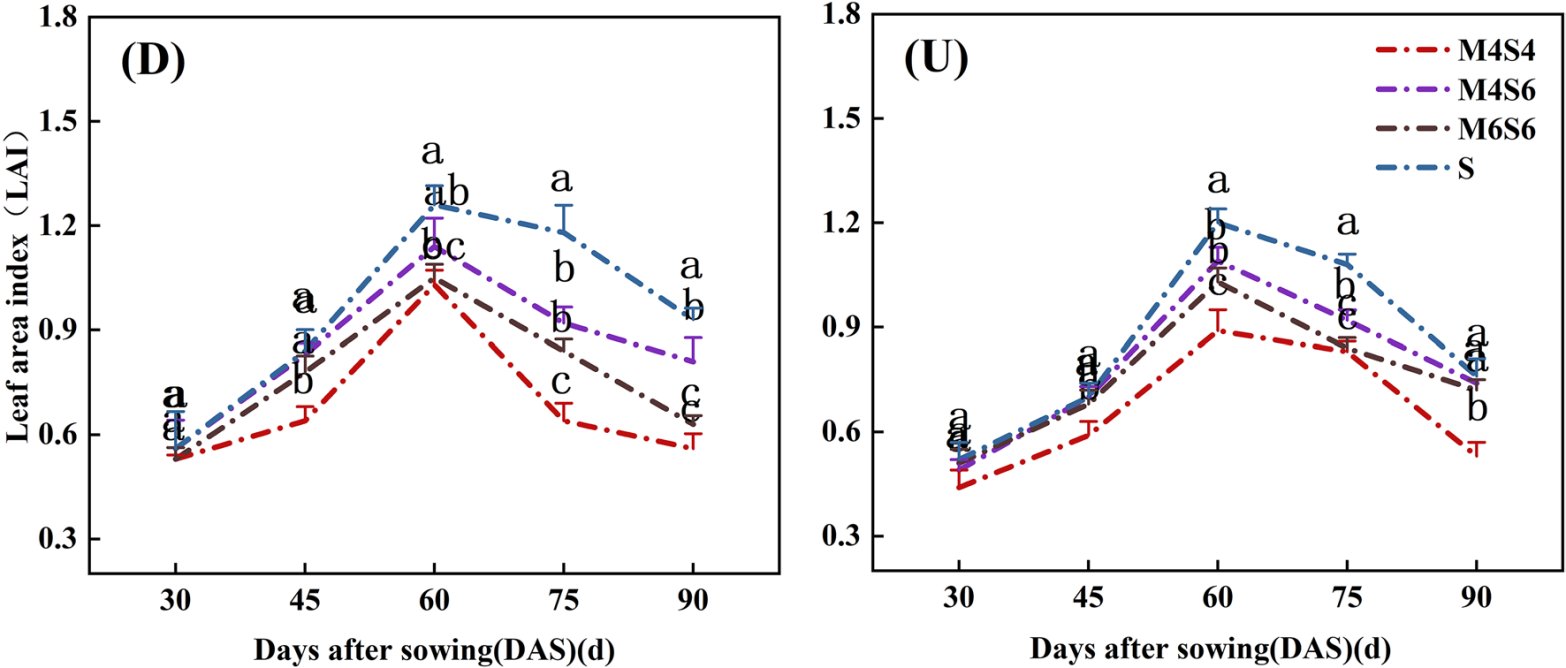
Leaf area indices of soybean under different intercropping patterns. *Note*: Different lowercase letters above the lines with the same colours are significantly different (p < 0.05). (D) Narrow-wide row pattern of soybean; (U) uniform-ridge pattern of soybean (the same applies to the following figures).

The leaf area index of soybean in the uniform-ridge and narrow-wide row patterns initially increased and subsequently decreased as the soybean crops matured after sowing, resulting in a single-peak curve. The maximum leaf area index was reached 60 days after sowing. The leaf area index at the three intercropping ratios was lower than that in the monoculture of soybean (S) (Fig. 1). The leaf area index of soybean was 14.81% lower in the four rows of maize and six rows of soybean in the narrow-wide row planting pattern (D-M4S6), 18.01% lower in the six rows of maize and six rows of soybean in the narrow-wide row planting pattern (D-M6S6), and 26.56% lower in the four rows of maize and four rows of soybean in the narrow-wide row planting pattern (D-M4S4) than in the soybean monoculture in the narrow-wide row planting pattern (D-S). Similarly, the leaf area indices in the four rows of maize and six rows of soybean in the uniform-ridge planting pattern (U-M4S6), six rows of maize and six rows of soybean in the uniform-ridge planting pattern (U-M6S6), and four rows of maize and four rows of soybean in the uniform-ridge planting pattern (U-M4S4) were 7.81%, 10.58%, and 23.19% lower, respectively, than those of the soybean monoculture in the uniform-ridge planting pattern (U-S). Under the three intercropping ratios, the leaf area indices of M6S6 and M4S6 were significantly greater than that of M4S4, and the leaf area index of the M4S6 treatment reached the highest value. The average of leaf area indices under the D-M4S6 and U-M4S6 were 6.7% greater than that under the M6S6 treatment and 10.11% greater than that under the M4S4 treatment. The leaf area index of soybean in the narrow-wide row planting pattern (D) was greater than that in the uniform-ridge planting pattern (U) for the same intercropping ratio. However, the differences were not statistically significant.

Table 1 shows that at 30–90 days after sowing, the intercropping ratio (MS), planting pattern (DU), and MS * DU had significant direct and interaction effects on the leaf area index of soybean at some growth stages. At 45–90 days after sowing, MS had a significant effect on the leaf area index of soybean. The DU had a significant effect on the leaf area index of soybean at 30 days, 45 days, 60 days and 90 days after sowing. At 75–90 days after sowing, MS * DU had a significant effect on the soybean leaf area index.

**Table 1:** Direct and interaction effects of planting pattern and intercropping ration on leaf area index.

Treatment	Leaf area index				
	30d	45d	60d	75d	90d
ANOVA					
Intercropping ratio (MS)	NS	**	**	**	**
Planting pattern (DU)	*	**	**	NS	**
MS*DU	NS	NS	NS	**	**

*NS* not significant.

*Significant at the 0.05 level; **significant at the 0.01 level.

### Photosynthetically active radiation

**Figure 2:**
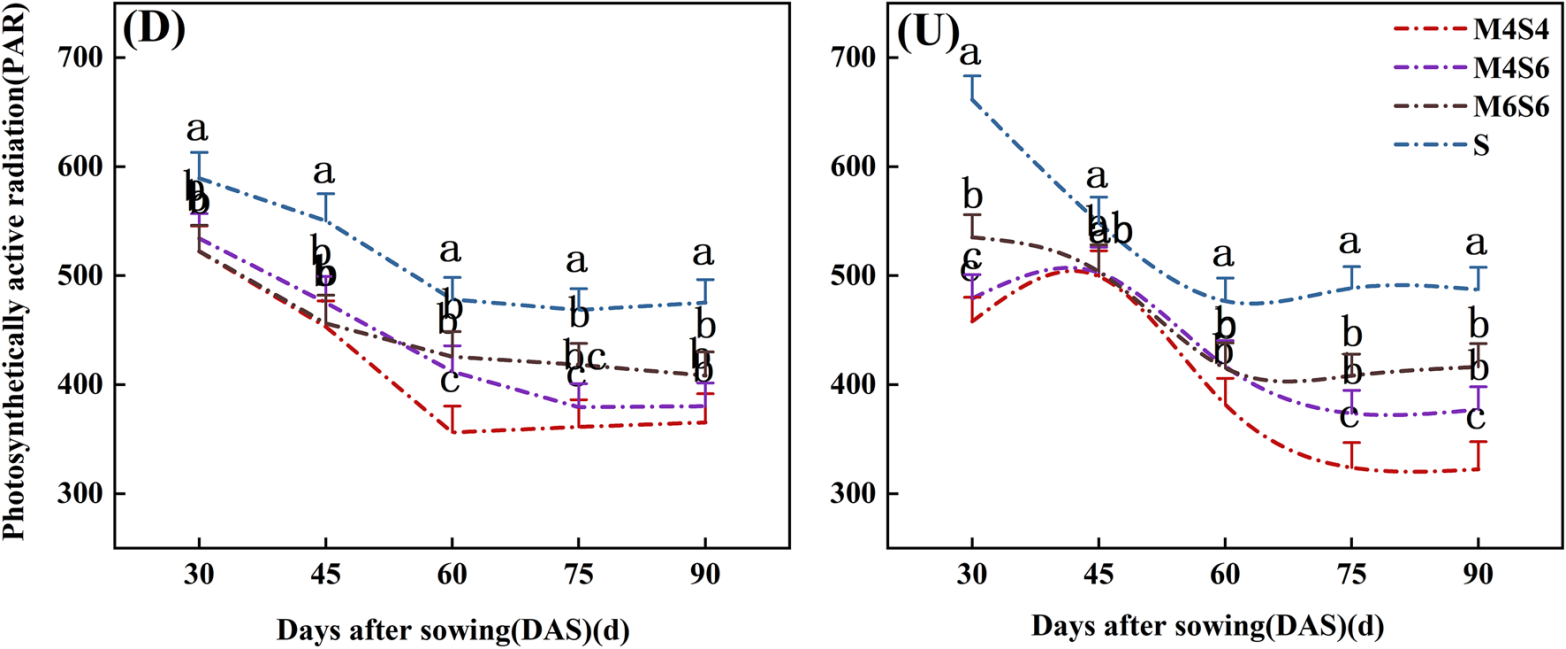
Photosynthetically active radiation of soybean with different intercropping patterns. *Note*: Different lowercase letters above the lines with the same colour are significantly different (p < 0.05).

The photosynthetically active radiation of soybean under two planting patterns decreased steadily over time (Fig. 2). For the narrow-wide row planting pattern (D), the photosynthetically active radiation under D-M6S6, D-M4S6, and D-M4S4 treatments was 13.14%, 15.17%, and 20.15% lower than that under D-S, respectively. Similarly, the photosynthetically active radiation under U-M6S6, U-M4S6, and U-M4S4 treatments was 14.22%, 18.92%, and 25.42% lower than that under U-S, respectively. When the same intercropping ratios were maintained, the average photosynthetically active radiation determined after sowing in the narrow-wide row planting pattern increased relative to that in the uniform-ridge planting pattern. Under the three intercropping ratios, the photosynthetically active radiation of M6S6 and M4S6 was significantly greater than that of M4S4, and that of the M6S6 treatment was greatest. The average of photosynthetically active radiation of the narrow-wide row planting pattern (D) and uniform- ridge planting pattern (U) was 5.81% greater than that under the M4S6 treatment and 12.58% greater than that under the M4S4 and M6S6 treatments.

Table 2 shows that at 30–90 days after sowing, the intercropping ratio (MS) had a significant effect on the photosynthetically active radiation, while the planting pattern (DU) had no significant effect. The DU had a significant effect on photosynthetically active radiation only at 45 days after sowing. From 45 to 90 days after sowing, MS * DU had a nonsignificant effect on photosynthetically active radiation of soybean.

**Table 2:** Direct and interaction effects and of planting pattern and intercropping ratio on photosynthetically active radiation.

Treatment	Photosynthetically active radiation				
	30d	45d	60d	75d	90d
ANOVA					
Intercropping ratio (MS)	**	**	**	**	**
Planting pattern (DU)	NS	**	NS	NS	NS
MS*DU	**	NS	NS	NS	NS

*NS* not significant.

*Significant at the 0.05 level; **significant at the 0.01 level.

### Light transmittance ratio

**Figure 3:**
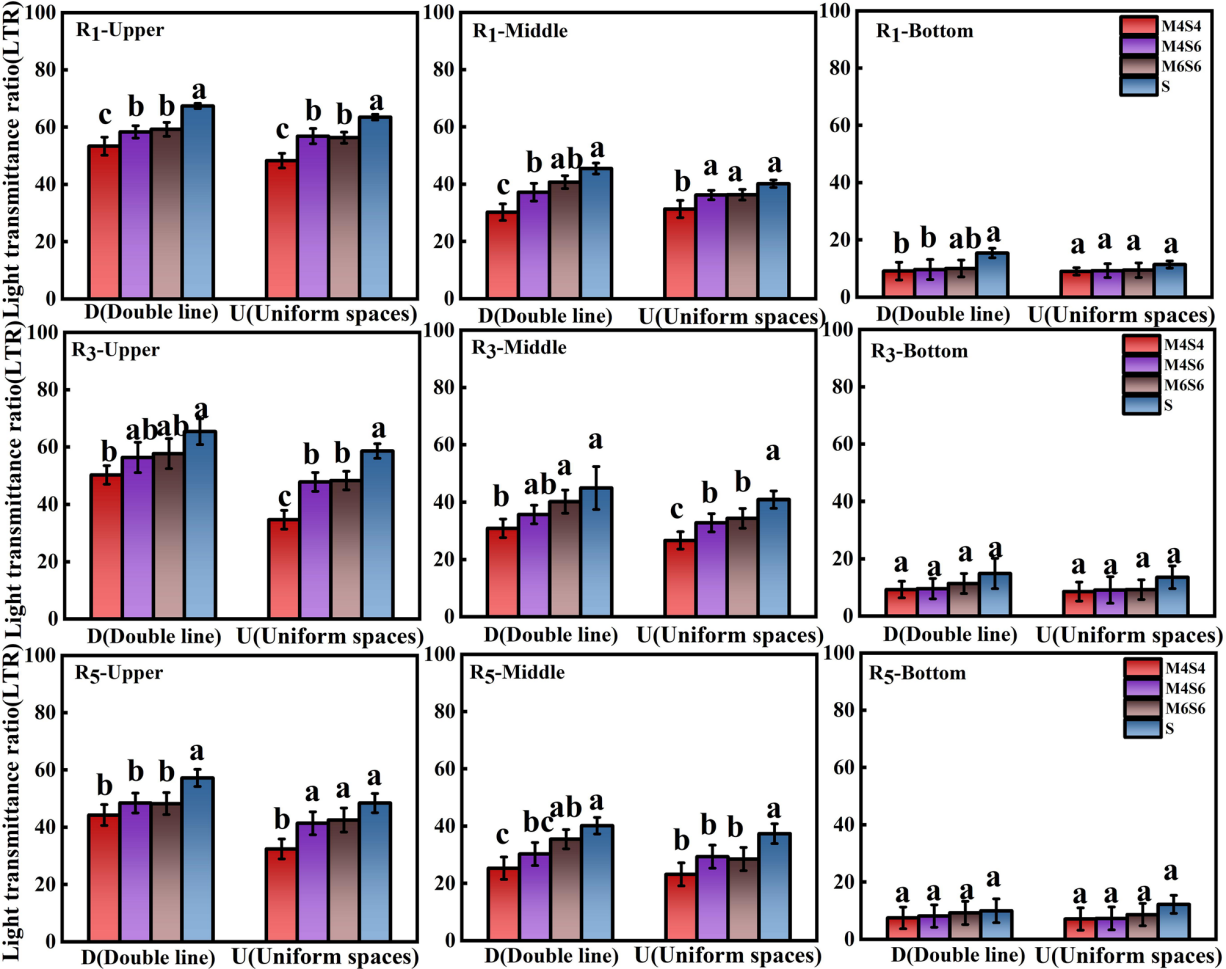
Light transmittance ratio during the R1, R3, and R5 stages under the different intercropping patterns. *Note*: Different lowercase letters above the bars with the same colours are significantly different (p < 0.05). The upper panel shows the average points on the upper leaves of each row of soybeans; the middle panel shows the average points on the middle leaves of each row of soybeans, and the bottom panel shows the average points on the bottom leaves of each row of soybeans. R1, initial flowering stage; R3, initial pod stage; R5, initial grain stage.

The light transmittance ratios of the bottom, middle, and upper leaves of the soybeans increased steadily during the R1, R3, and R5 stages. Compared to the monoculture of soybean (Fig. 3), the average light transmittance ratio of soybean in the R1 stage decreased by 31.67% (4:4), 22.98% (4:6), and 19.13% (6:6) in the narrow-wide-row pattern and by 22.27% (4:4), 12.98% (4:6), and 12.72% (6:6) in the uniform-ridge pattern. Among the different intercropping ratios tested during the R3 stage, the treatment 6:6 intercropping ratio had a greater mean value than did the 4:4 and 4:6 intercropping ratios (Fig. 3). The light transmittance ratios at the 6:6 intercropping ratio were 12.8% (bottom leaves), 22.75% (middle leaves), and 20.55% (upper leaves) greater than those at the 4:4 ratio, and 8.49% (bottom leaves), 7.8 (middle leaves), and 1.68% (upper leaves) greater than those at the 4:6 ratio. The light transmittance ratio of soybean leaves was lower during the R5 stage than during the other stages. Similarly, the light transmittance ratios for the D-M6S6 were 18.52% and 13.3% greater than those for the D-M4S4 and D-M4S6, respectively, and 20.15% and 4.78% greater than those for the U-M4S4 and U-M4S6, respectively, for the uniform-ridge pattern. However, the average light transmittance ratio did not differ significantly between the different patterns under the same intercropping ratio.

During the R1, R3, and R5 stages of soybean growth, intercropping ratio (MS) showed a significant effect on all soybean canopy leaves; however, during the R3 and R5 stages, the effect on the bottom leaves of soybean was not significant. The planting pattern (DU) had no significant effect on the bottom leaves of soybean at the R1, R3, or R5 stages but had a significant effect on the middle and upper leaves. The effect of MS * DU on soybean light transmittance was not significant during the R1, R3, or R5 stages (Table 3).

**Table 3:** Direct and interaction effects of planting pattern and intercropping on the light transmittance ratio.

Treatment	Light transmittance ratio								
	R1			R3			R5		
	Upper	Middle	Bottom	Upper	Middle	Bottom	Upper	Middle	Bottom
ANOVA									
Intercropping (MS)	**	**	*	**	**	NS	**	**	NS
Planting pattern (DU)	**	*	NS	*	*	NS	**	*	NS
MS*DU	NS	NS	NS	NS	NS	NS	NS	NS	NS

*NS* not significant.

*Significant at the 0.05 level; **significant at the 0.01 level.

### Stem bending resistance of soybean

The stem bending resistance of the narrow-wide-row planting pattern (D) was significantly greater than that of the uniform-ridge planting pattern (U) under the same intercropping ratio (Table 4). Compared to that under U-M4S6, the average soybean stem bending resistance under D-M4S6 increased by 16.49% (3rd node), 7.24% (4th node), and 2.35% (5th node). At a 4:6 ratio, the average stem bending resistance of soybean was 15.27% (3rd node), 11.21% (4th node), and 9.67% (5th node) greater than that at a 4:4 ratio, and 2.61% (3rd node), 1.59% (4th node), and 1.99% (5th node) greater than that at a 6:6 ratio.

**Table 4:** Stem bending resistance of the different internodes of soybean plants under the different intercropping patterns.

Treatment	R1			R3			R5		
	3rd node	4th node	5th node	3rd node	4th node	5th node	3rd node	4th node	5th node
D (double line)									
M4S4	273.5 ± 16.2cd	273.7 ± 15.8bc	215.3 ± 16.3cd	298.9 ± 12.8cd	285.9 ± 14.1bc	228.7 ± 10.3c	279.9 ± 9.0c	288.9 ± 11.9cd	226.1 ± 7.0cd
M4S6	324.5 ± 12.4a	313.9 ± 14.5a	245.2 ± 11.5ab	345.6 ± 11.7a	324.0 ± 12.8a	254.5 ± 14.7a	335.6 ± 13.6b	317.7 ± 9.8ab	242.3 ± 11.7abc
M6S6	322.2 ± 12.0a	312.3 ± 8.3a	245.1 ± 13.0ab	333.8 ± 12.2ab	323.7 ± 14.4a	249.8 ± 12.5ab	323.2 ± 12.3b	304.1 ± 11.3bc	232.2 ± 9.9bcd
S	328.6 ± 15.3a	322.5 ± 14.1a	267.4 ± 13.3a	348.3 ± 11.2a	337.1 ± 13.5a	266.9 ± 10.6a	357.0 ± 10.0a	325.6 ± 11.4a	246.2 ± 9.4ab
U (uniform space)									
M4S4	266.2 ± 12.5d	257.8 ± 11.8c	198.2 ± 13.0d	268.1 ± 12.1e	265.4 ± 10.9c	234.2 ± 13.4bc	211.3 ± 11.3d	277.9 ± 12.9d	220.12 ± 9.7d
M4S6	315 ± 11.7ab	282.8 ± 9.4b	225.6 ± 11.3bc	317.7 ± 7.1bc	297.0 ± 7.7b	259.1 ± 10.9a	295.0 ± 7.7c	306.6 ± 10.3abc	240.3 ± 8.5bc
M6S6	295.4 ± 12.2bc	279 ± 18.8bc	223.7 ± 9.2bc	288.2 ± 8.4d	288.6 ± 10.5b	256.3 ± 6.1a	289.1 ± 8.8c	300.2 ± 8.6bc	237.9 ± 6.5bc
S	319.9 ± 13.1a	283.6 ± 8.8b	244.7 ± 9.5ab	335.8 ± 13.7ab	299.4 ± 9.9b	267.9 ± 8.5a	329.9 ± 9.1b	314.1 ± 7.1ab	257.2 ± 7.6a
ANOVA									
Intercropping ratio (MS)	**	**	**	**	**	**	**	**	**
Planting pattern (DU)	*	**	**	**	**	NS	**	*	NS
MS*DU	NS	NS	NS	*	NS	NS	*	NS	*

Values followed by different letters in the same column are significantly different at the 5% level. R1, R3, R5 refer to the initial flowering stage, initial pod stage and initial grain stage. 3rd node, 4th node and 5th node refer to the third, fourth and fifth nodes of soybean.

NS, *, ** indicate nonsignificant or significant at the 0.05 or 0.01 level, respectively.

At the R1, R3, and R5 stages of soybean growth, the intercropping ratio (MS) had a significant effect on stem bending resistance. The planting pattern (DU) also had a significant effect on the stem bending resistance of soybean at the R1, R3, and R5 stages; however, at the R3 stage on the 4th node, the effect was not significant. MS * DU had a significant effect only on the 3rd node at the R3 stage and on the 4th and 5th nodes at the R5 stage. The narrow-wide row planting pattern greatly improved the soybean stem bending resistance. The stem bending resistance of the 3rd node was the highest throughout the growth period. The narrow-wide row planting pattern significantly improved the stem bending resistance of the 3rd and 4th nodes of soybean but had little effect on that of the 5th node.

### Cellulose, hemicellulose, and lignin contents of soybean stem

The cellulose, hemicellulose and lignin contents under the narrow-wide row planting pattern (D) were greater than those under the uniform-ridge planting pattern (U) at the same intercropping ratio (Table 5). Under the three intercropping ratios, the cellulose, hemicellulose and lignin contents of M6S6 and M4S6 were significantly greater than those of M4S4, and those of the M4S6 treatment were greatest. Compared to that under U-M4S6, the cellulose content under D-M4S6 increased by 6.21% (R1 stage), 1.52% (R3 stage), and 2.16% (R5 stage). At the 4:6 ratio, the average cellulose content of soybean was 19.05% (R1 stage), 8.14% (R3 stage), and 6.09% (R5 stage) higher than that at the 4:4 ratio, and the average cellulose content was 3.55% (R1 stage), 1.97% (R3 stage), and 2.08% (R5 stage) higher than that at the 6:6 ratio. During the R1, R3, and R5 stages of soybean growth, the intercropping ratio (MS) had a significant effect on the cellulose content. The planting pattern (DU) had a significant effect on the cellulose content in the R1 stage. MS * DU had no significant effect on the cellulose content at the R1, R3 or R5 stages.

**Table 5:** Cellulose, hemicellulose and lignin contents of the different internodes of soybean plants under the different intercropping patterns.

Treatment	Cellulose content(mg g^-1^)			Hemicellulose content(mg g^-1^)			Lignin content(mg g^-1^)		
	R1	R3	R5	R1	R3	R5	R1	R3	R5
D (double line)									
M4S4	36.3 ± 2.9 c	42.4 ± 1.6bc	43.9 ± 2.8bc	10.8 ± 0.7c	11.8 ± 2.3c	12.2 ± 1.6bc	20.9 ± 0.7e	26.1 ± 0.9cd	23.1 ± 1.1b
M4S6	45.1 ± 2.7ab	45.8 ± 0.8abc	46.3 ± 2.1abc	13.8 ± 1.1ab	16.5 ± 0.3a	14.5 ± 1.8ab	26.1 ± 1.0b	29.4 ± 1.0ab	25.8 ± 1.3a
M6S6	43.5 ± 3.0ab	44.6 ± 2.0abc	45.5 ± 2.3abc	13.6 ± 1.1ab	15.8 ± 1.7a	14.1 ± 2.1ab	25.3 ± 1.0bc	28.5 ± 1.1ab	25.5 ± 0.9ab
S	45.7 ± 1.1a	47.0 ± 2.2a	48.5 ± 2.4a	14.5 ± 0.8a	16.8 ± 0.7a	14.8 ± 2.0a	28.9 ± 1.1a	31.1 ± 1.2a	26.4 ± 0.6a
U (uniform)									
M4S4	34.4 ± 2.8c	41.1 ± 1.5.c	42.1 ± 1.7c	8.9 ± 0.6d	12.3 ± 1.3bc	11.4 ± 1.2c	22.6 ± 0.8de	23.6 ± 0.7d	25.5 ± 1.0ab
M4S6	42.3 ± 2.1ab	45.1 ± 3.0abc	45.3 ± 1.8abc	12.9 ± 1.0ab	15.1 ± 1.0ab	13.2 ± 1.7bc	24.1 ± 1.2bcd	28.6 ± 1.2ab	27.2 ± 1.1a
M6S6	40.8 ± 1.5b	44.5 ± 2.5abc	44.2 ± 2.8abc	12.4 ± 1.0b	14.9 ± 3.0ab	12.8 ± 3.0b	23.1 ± 1.1cde	27.9 ± 1.2bc	26.1 ± 1.2ab
S	43.9 ± 1.7ab	46.3 ± 5.2ab	46.7 ± 1.5ab	13.2 ± 1.0ab	15.3 ± 2.4ab	13.7 ± 1.2abc	25.4 ± 0.7bc	28.8 ± 1.2ab	26.7 ± 1.4a
ANOVA									
Intercropping ratio (MS)	**	*	*	**	**	NS	**	**	**
Planting pattern (DU)	**	NS	NS	**	NS	NS	**	**	*
MS*DU	NS	NS	NS	NS	NS	NS	**	NS	NS

Values followed by different letters in the same column are significantly different at the 5% level.

NS, *, ** indicate nonsignificance or significant at the 0.05 or 0.01 level, respectively.

Compared with that under U-M4S6, the hemicellulose content under D-M4S6 increased by 6.52% (R1 stage), 8.49% (R3 stage), and 8.97% (R5 stage). At the 4:6 ratio, the average hemicellulose content of soybean was 26.35% (R1 stage), 23.51% (R3 stage), and 14.81% (R5 stage) greater than that at the 4:4 ratio, and the average hemicellulose content was 2.66% (R1 stage), 2.78% (R3 stage), and 2.89% (R5 stage) greater than that at the 6:6 ratio. During the R1 and R3 stages, the intercropping ratio (MS) had a significant effect on the hemicellulose content. The planting pattern (DU) had a significant effect on the cellulose content in the R1 stage. MS * DU had no significant effect on the hemicellulose content of soybean at the R1, R3 or R5 stages.

Compared to that under U-M4S6, the lignin content under D-M4S6 increased by 7.66% (R1 stage) and 2.72% (R3 stage). At the 4:6 ratio, the average lignin content of soybean was 13.06% (R1 stage), 14.31% (R3 stage), and 8.36% (R5 stage) greater than that at the 4:4 ratio, and 3.60% (R1 stage), 2.75% (R3 stage), and 2.60% (R5 stage) higher than that at the 6:6 ratio. During the R1, R3 and R5 stages, the intercropping ratio (MS) and planting pattern (DU) had significant effects on the lignin content. At the R1 stage, MS * DU had a significant effect on lignin content.

### PAL, TAL and CAD activities in soybean stems

**Figure 4:**
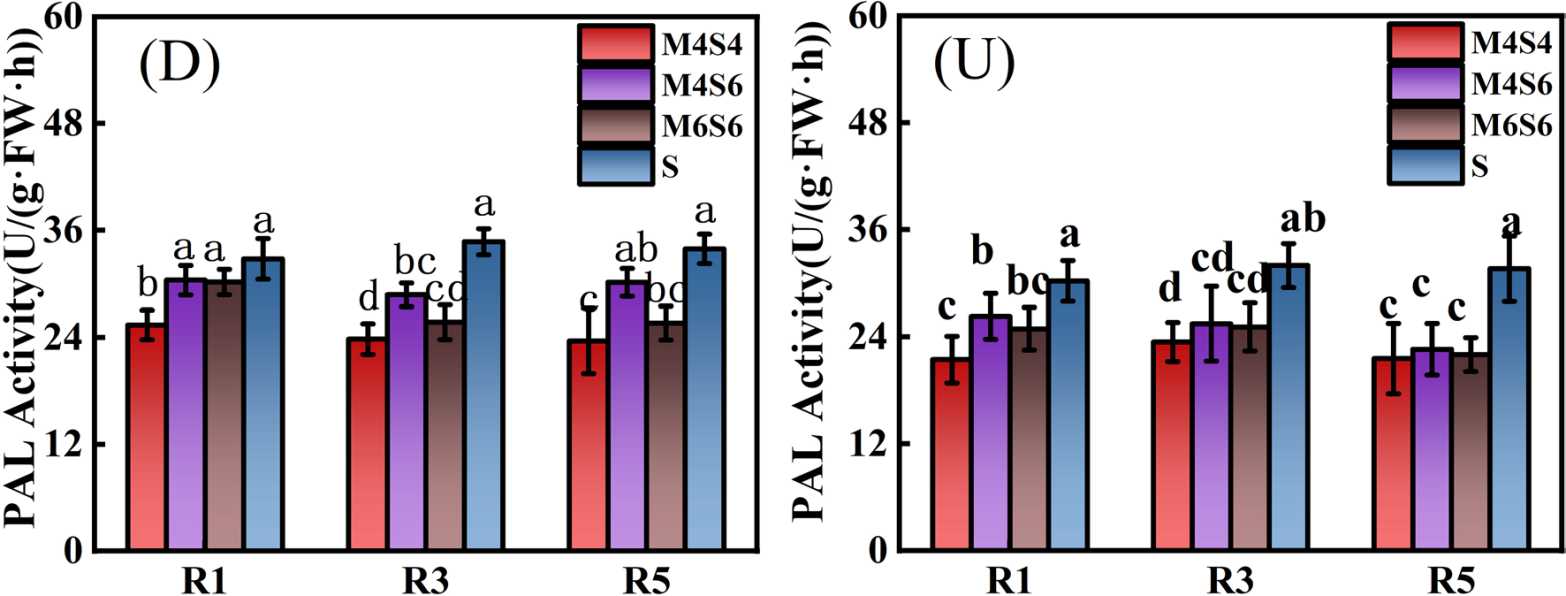
PAL activity of soybean stems in the R1, R3 and R5 stages under different intercropping patterns. *Note*: Different lowercase letters above the bars with the same colour are significantly different (p < 0.05).

Figure 4 shows the PAL activity of soybean stems. The soybean monoculture in the narrow-wide row planting pattern (D-S) resulted in the highest mean values of 32.79 U/(g·FW·h) (R1 stage), 34.71 U/(g·FW·h) (R3 stage), and 33.91 U/(g·FW·h) (R5 stage). The PAL activity in the DS treatment was 22.6% (R1 stage), 31.4% (R3 stage), and 30.3% (R5 stage) greater than that in the D-M4S4 treatment. The PAL activity in the DS treatment was 7.29% (R1 stage), 17.14% (R3 stage), and 11.06% (R5 stage) greater than that in the D-M4S6 treatment, and 7.93% (R1 stage), 25.96% (R3 stage), and 24.51% (R5 stage) greater than that in the D-M6S6 treatment. Generally, the PAL activity in the D-M4S6 treatment was greater than that in the D-M4S4 and D-M6S6 treatments. In addition, the PAL activity in the D-M4S6 treatment was 13.52% (R1 stage), 11.47% (R3 stage), and 25.03% (R5 stage) greater than that in the U-M4S6 treatment. The PAL activity in the narrow-wide row pattern (D) was significantly greater than that in the uniform-ridge pattern (U). Under the three intercropping ratios, the PAL activity in the M6S6 and M4S6 treatments was significantly greater than that in the M4S4 treatment, and the PAL activity in the M4S6 treatment reached its highest value. The average PAL activities of D and U were 16.48% (R1 stage), 17.21% (R3 stage), 21.71% (R5 stage) greater than that of the M4S4 treatment and 0.69% (R1 stage), 10.63% (R3 stage), 15.12% (R5 stage) greater than that of the M6S6 and M4S6 treatments.

**Figure 5:**
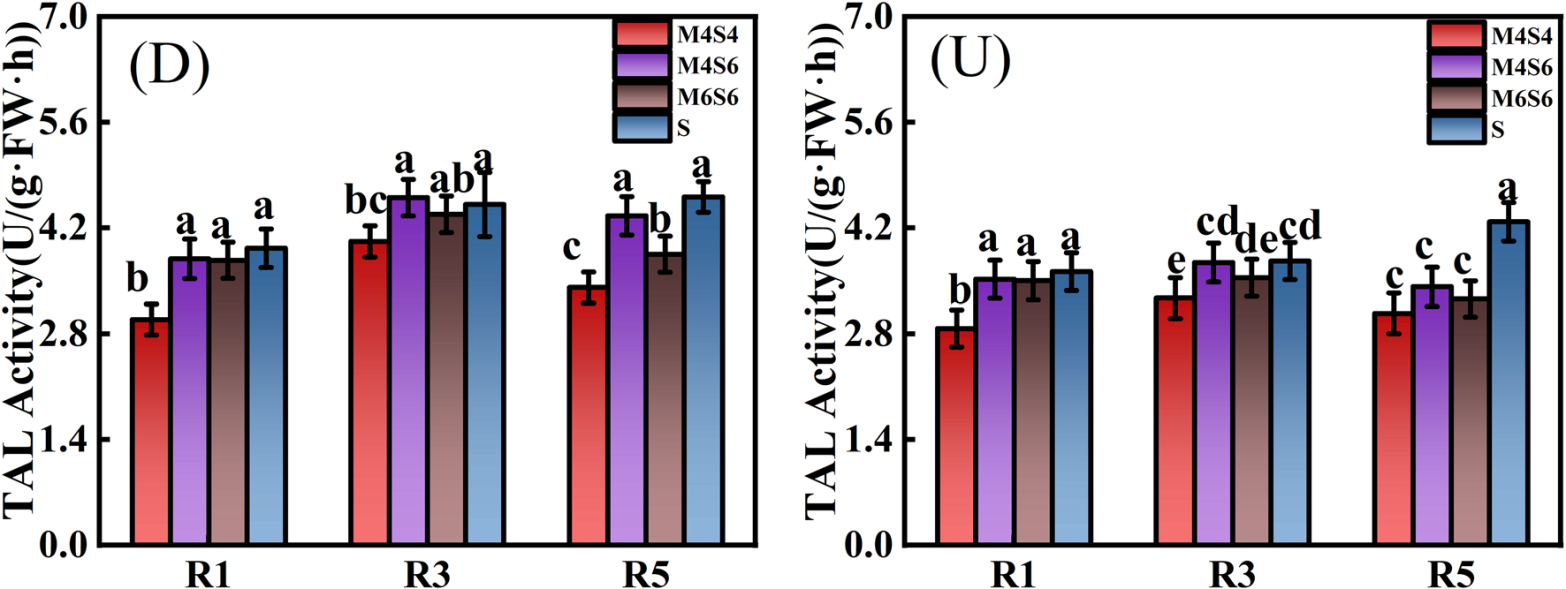
TAL activity of soybean stems in the R1, R3 and R5 stages under different intercropping patterns. *Note*: Different lowercase letters above the bars with the same colour are significantly different (P < 0.05).

Figure 5 shows the TAL activity of soybean stems. In the R3 and R5 stages, TAL activity was considerably greater in the narrow-wide-row planting pattern (D) than in the uniform-ridge planting pattern (U). In the R1 stage, there was no significant difference in TAL activity between the narrow-wide row and uniform-ridge patterns. Compared with that of D-S, the average TAL activity of soybean stems decreased by 20.27% (D-M4S4), 2.33% (D-M4S6), and 7.81% (D-M6S6). Compared with that in the U-S treatment, the average TAL activity in the soybean stem decreased by 20.67% (U-M4S4), 7.79% (U-M4S6), and 10.98% (U-M6S6). Under the three intercropping ratios, the TAL activity of M6S6 and M4S6 was significantly greater than that of M4S4, and that of the M4S6 treatment reached its highest value. The average of TAL activity under the (D) and (U) patterns was 19.75% (R1 stage), 12.6% (R3 stage), and 15.95% (R5 stage) greater than that of the M4S4 treatment and 0.55% (R1 stage), 5.06% (R3 stage), and 8.18% (R5 stage) greater than that of the M6S6 treatment under the M4S6 treatment.

**Figure 6:**
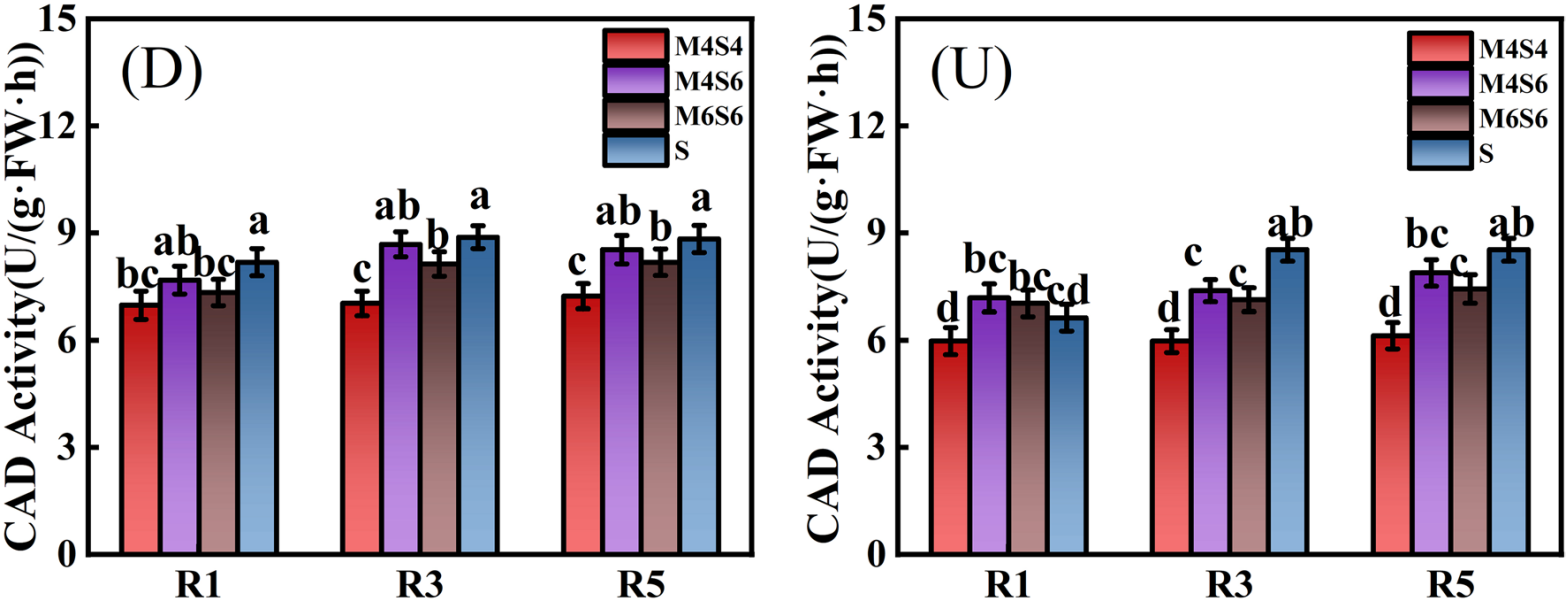
CAD activity of soybean stems in the R1, R3 and R5 stages under different intercropping patterns. *Note*: Different lowercase letters above the bars with the same colours are significantly different (P < 0.05).

Figure 6 shows the CAD activity of soybean stems. The average CAD activity of soybean in the D-S treatment was 17.87% greater than that in the D-M4S4 treatment, 3.92% greater than that in the D-M4S6 treatment, and 8.73% greater than that in the D-M6S6 treatment. The average CAD activity of soybean in the U-S treatment was 20.61% greater than that in the U-M4S4 treatment, 10.55% greater than that in the U-M4S6 and 14.65% greater than that in the U-M6S6 treatment. The CAD activity was significantly greater in the narrow-wide row planting pattern (D) than in the uniform-ridge planting pattern (U). Under the three intercropping ratios, the CAD activity in the M4S6 treatment was significantly greater than that in the M4S4 and M6S6 treatments. The average CAD activity under the (D) and (U) patterns was 12.91% (R1 stage), 18.9% (R3 stage), and 18.72% (R5 stage) greater than that of the M4S4 treatment, and 3.32% (R1 stage), 4.86% (R3 stage), and 4.91% (R5 stage) greater than that of the M6S6 and M4S6 treatments.

During the R1, R3 and R5 stages of soybean, intercropping (MS) had a effectively significant effect on PAL, TAL, and CAD activity in soybean stems. Planting pattern (DU) had a significant effect on PAL activity in the R1 and R5 stages, but had no significant effect on PAL activity in soybean in the R3 stage. DU had a significant effect on TAL and CAD activity in the R1, R3, and R5 stages. MS * DU had no significant effect on PAL, TAL, or CAD activity and only had a significant effect on CAD activity in the R1 stage (Table 6).

**Table 6:** Direct and interaction effects of planting pattern and intercropping on the PAL, TAL and CAD activities of soybean.

Treatment	PAL Activity			TAL Activity			CAD Activity		
	R1	R3	R5	R1	R3	R5	R1	R3	R5
ANOVA									
Intercropping ratio(MS)	**	**	**	**	**	**	**	**	**
Planting pattern(DU)	*	NS	**	*	**	**	**	**	**
MS * DU	NS	NS	NS	NS	NS	NS	*	NS	NS

*NS* not significant.

*Significant at the 0.05 level; **significant at the 0.01 level.

### Soybean grain yield

For the same intercropping ratio, the compound yield in the narrow-wide row pattern was significantly greater than that in the uniform-ridge pattern; for example, the compound yield of D-M6S6 increased by 5.97% compared with that of U-M6S6, the compound yield of D-M4S6 increased by 5.98% compared with that of U-M4S6, and the compound yield of D-M4S4 increased by 5.89% compared with that of U-M4S4 (Table 7). When grown under the same planting pattern, the compound yields of M6S6 and M4S6 were significantly greater than that of M4S4; for example, the compound yield of D-M6S6 increased by 3.37% compared with that of D-M4S4, and the compound yield of D-M4S6 increased by 2.66% compared with that of D-M4S4. However, there was no significant difference in compound yield between M6S6 and M4S6, and D-M6S6 and D-M4S6 had the same LER value (1.16).

**Figure 7:**
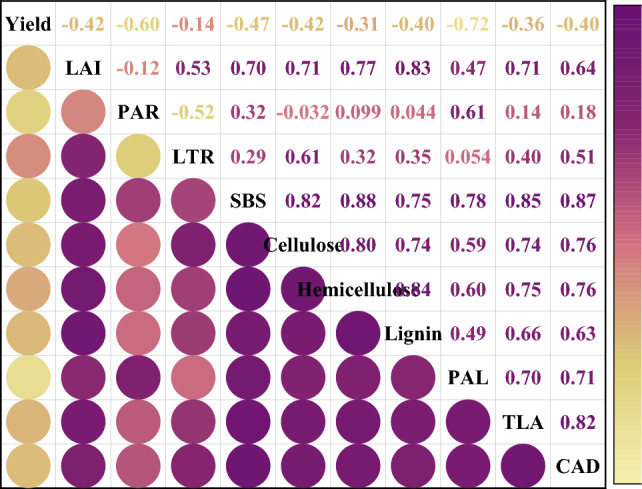
Pearson correlations between yield and the photosynthetic and bending resistance characteristics. *Note*: The depth of colour denotes significance at the p < 0.05 probability level, the dark colour represents significance, and the light colour represents nonsignificance. *LAI* leaf area index, *PAR* photosynthetically active radiation, *LTR* light transmittance ratio, *SBR* stem bending resistance.

### Correlations between yield, canopy photosynthesis, stem bending resistance, and related enzyme activities

We used a network matrix to examine the correlation between yield, LAI, PAR, LTR, stem bending resistance, cellulose, hemicellulose, lignin, and PAL, TAL, and CAD activities (Fig. 7). The soybean yield was negatively correlated with the other indicators. There was a negative correlation between the leaf area index and photosynthetically active radiation. However, stem bending resistance was positively correlated with cellulose, hemicellulose, lignin, PAL, TAL, and CAD activities.

**Figure 8:**
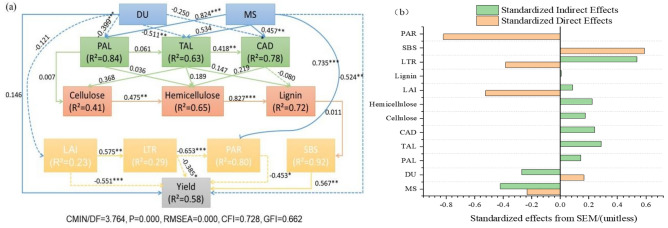
Planting patterns and intercropping ratios using structural equation modeling (**a**) and standardized effects (**b**). *Note*: DU: planting pattern (including narrow-wide-row pattern and uniform-ridge pattern); MS: different intercropping ratio; LAI: leaf area index; PAR: photosynthetically active radiation; LTR: light transmittance ratio; PAL: phenylalanine ammonia-lyase; TAL: tyrosine ammonia-lyase; CAD: cinnamyl alcohol dehydrogenase; SBS: stem bending resistance. The solid arrows represent a positive correlation, and dotted arrows represent a negative correlation.

A structural equation model (SEM) indicated that of the planting pattern and intercropping ratio accounted for 58% of the effects on yield, as shown in Fig. 8a. The main explanatory factors included PAL, TAL, CAD, cellulose, hemicellulose, lignin, LAI, PAR and LTR, and the overall fit of the model was acceptable (CMIN/DF = 3.67, p = 0.000, RMSEA = 0.00, CFI = 0.728, GFI = 0.662). The SEM results showed that the intercropping ratio had a significant positive correlation with the activity of PAL, TAL, and CAD, while the planting pattern showed a significant negative correlation. The activities of the three enzymes were positively correlated with the contents of soybean cellulose, lignin and hemicellulose and were also positively correlated with stem bending resistance.

The results of standardized effects (b) showed that the intercropping ratio, planting pattern, photosynthetically active radiation, light transmittance, leaf area index and stem bending resistance had significant direct effects on soybean yield, while cellulose, hemicellulose, lignin, PAL, TAL, and CAD activity and stem bending resistance had significant indirect effects on soybean yield. Combining the direct and indirect effects, it was found that the planting pattern and intercropping ratio significantly affected the enzyme activity, promoted the synthesis of cellulose and lignin, and improved the stem bending resistance of soybean plants. However, due to the negative correlation between the planting pattern and soybean canopy photosynthesis, the soybean yield decreased.

## Discussion

Light is an essential factor for crop photosynthesis^22^. Crops perform photosynthesis using the chloroplasts on their leaves, converting solar energy into chemical energy; the size of the leaf area index directly affects the area of crop photosynthesis^23^. Planting a combination of C3 and C4 crops can maximize area utilization and provide complementary benefits; however, the impact of maize shading must not be overlooked^24^. Our findings showed that maize‒soybean intercropping changed the leaf area index, photosynthetically active radiation, and light transmittance ratio of soybean and that these values significantly decreased compared to those under soybean monoculture, which is consistent with previous findings^25,26^. The row ratio structure alters the distribution of light energy in maize and soybean populations^27^. In our study, the narrow-wide row pattern (80 cm–50 cm) increased the photosynthetically active radiation of soybean significantly more than did the uniform-ridge pattern (65 cm) because the change in ridge spacing not only improved the light transmittance ratio of soybean but also increased the photosynthetic area of crops. The average photosynthetically active and light transmittance ratios of D-M6S6 were significantly greater than those of than the other soybean treatments.

The soybean stem is a vital component for plant maintenance, and lodging frequently occurs in the stem internodes. Therefore, the stem bending resistance of soybean plants is closely related to lodging, which indirectly affects soybean yield^28^. Previous studies have shown that intercropping reduces soybean stem bending resistance and increases the likelihood of soybean lodging^29^, which is consistent with our findings. Compared with monocultures, intercropping reduces soybean stem bending resistance to varying degrees. Under the various intercropping ratios, the stem bending resistance of soybean plants tended to decrease in the following order: S > M4S6 > M6S6 > M4S4. The reason for this could be that the strip intercropping of soybean was shaded by maize, which resulted in a reduced leaf area index and prevented soybean plants from accumulating sufficient organic matter, resulting in the inhibition of stem growth and germination and a decrease in stem bending resistance (Fig. 3). However, by changing the row spacing and improving the microenvironment of the population, more organic matter can be transferred to the stems in soybean, which can effectively improve the bending resistance of soybean stems and lodging^30^. The narrow-wide row pattern considerably increased soybean stem bending resistance in our study by increasing the row spacing, allowing the soybean plants to have a larger light area, accumulate more organic matter, and transfer it to the stems.

**Figure 9:**
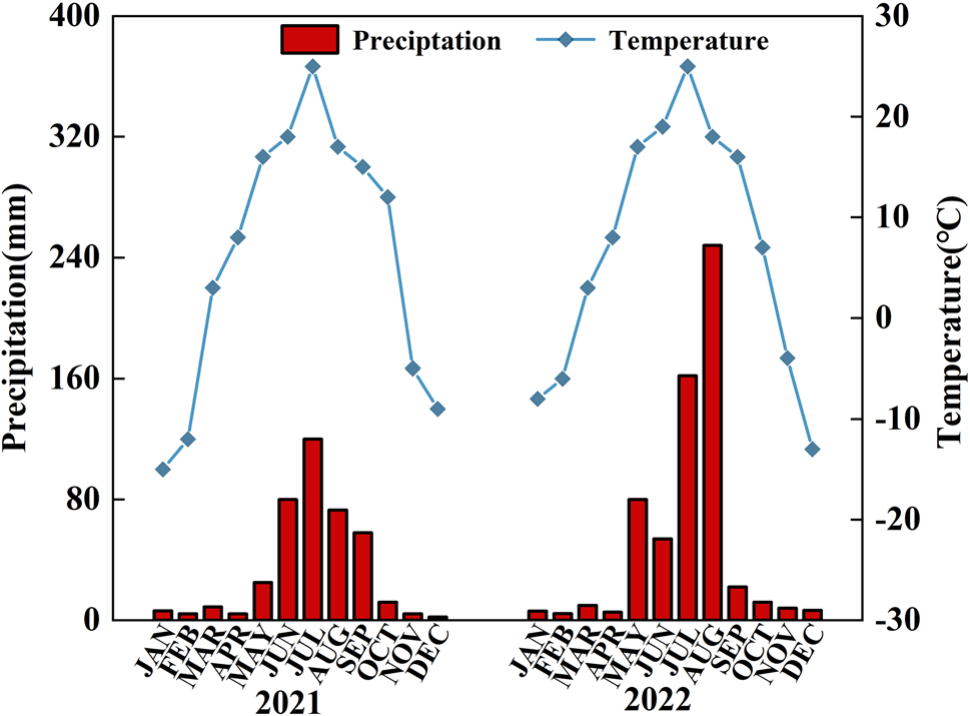
Average temperature and precipitation from May to October of 2021 and 2022.

Cellulose, hemicellulose, and lignin are significant components of the cell wall. Plant cell walls with strong fiber structures can provide mechanical support to plants^31^. Previous studies have shown that as the cellulose content of the soybean stems decreases, plants become more prone to lodging^29,32,33^. Cellulose, hemicellulose, and lignin contents are positively associated with stem bending resistance^34,35^. This finding is consistent with the results of our study. Figure 9 shows that the cellulose, hemicellulose, and lignin contents were positively related to stem bending resistance in our study, which enhanced soybean lodging. Table 5 shows the cellulose, hemicellulose, and lignin contents. These contents were found to be greatest in M4S6 at various intercropping ratios, which was consistent with the stem bending resistance results. Compared with the uniform-ridge planting pattern, the narrow-wide-row pattern improved the contents of cellulose, hemicellulose, and lignin, which could have resulted from an increase in the row spacing of 80 cm. This reduces the shading effect on soybean, resulting in increased cellulose, hemicellulose, and lignin accumulation, thereby enhancing the bending resistance of the soybean stem. Simultaneously, as crops mature, the degree of shading becomes less pronounced in the later growth stages. The cellulose content peaked in the R5 stage, whereas the hemicellulose and lignin contents peaked in the R3 stage.

**Table 7:** Comparison of maize and soybean yields and LER.

Treatment	Yield of soybean strip (kg ha^−1^)	Yield of maize strip (kg ha^−1^)	Compound yield of soybean (kg ha^−1^)	Compound yield of maize (kg ha^−1^)	Compound yield (kg ha^−1^)	LER
D (double lines)						
M4S4	2274.0 ± 121.3c	16,550.6 ± 282.7b	1137.0 ± 60.6c	8275.3 ± 141.4b	9412.3 ± 135.2cd	1.12
M4S6	2370.8 ± 125.6bc	16,968.4 ± 274.1ab	1185.4 ± 62.8bc	8484.2 ± 137.1ab	9669.6 ± 188.7c	1.16
M6S6	2369.6 ± 132.4bc	17,111.9 ± 256.3a	1184.8 ± 66.2bc	8555.9 ± 128.2a	9740.8 ± 190.0c	1.16
S	2626.0 ± 130.2a	–	2626.0 ± 130.2a	–	2626.0 ± 130.2f.	–
M	–	11,999.5 ± 268.8e	–	11,999.5 ± 268.8e	11,999.5 ± 268.8a	–
U (uniform spaces)						
M4S4	2181.7 ± 128.8c	15,533.4 ± 289.2d	1090.9 ± 64.4c	7766.7 ± 144.6d	8857.6 ± 183.8e	1.12
M4S6	2245.8 ± 135.1c	15,935.1 ± 276.3cd	1122.9 ± 67.6c	7967.5 ± 138.2cd	9090.4 ± 182.5de	1.15
M6S6	2257.8 ± 128.6c	16,060.7 ± 288.6c	1128.7 ± 64.3c	8030.3 ± 144.3c	9159.1 ± 207.3de	1.16
S	2576.2 ± 121.7ab	–	2601.2 ± 60.9ab	–	2601.2 ± 121.8f.	–
M	–	11,105.2 ± 276.8f.	–	11,105.2 ± 138.4f.	11,105.2 ± 276.8b	–
ANOVA						
Intercropping ratio(MS)	NS	NS	NS	NS	NS	
Planting pattern(DU)	NS	NS	NS	NS	NS	
MS * DU	**	**	**	**	**	

Values followed by different letters in the same column are significantly different at the 5% level.

The activities of the PAL, TAL, and CAD enzymes are positively related to the content of lignin and cellulose; enhanced PAL and CAD activities may lead to an increase in lignin content in the stem, which can serve as an essential indicator of lodging resistance^36,37^. Correlation analysis revealed a positive relationship between PAL, TAL, and CAD enzyme activities and cellulose, hemicellulose, and lignin under various intercropping ratios and planting patterns (Fig. 8). Our findings also demonstrated that the activity of these enzymes in the lignin synthesis pathway decreased under various intercropping ratios compared with that in a soybean monoculture. During the symbiotic period, plants compete for light because of the intercropping of maize and soybeans. Soybeans being a C3 crop, has lower enzyme activity, making it more susceptible to lodging^38^. However, changing the row spacing can substantially alleviate the competition for light between maize and soybean. Our findings revealed that the D-M4S6 treatment significantly enhanced the PAL, TAL, and CAD activities (Figs. 4, 5, 6). It also causes a significant increase in lignin content, which affects soybean stem bending resistance. This finding is similar to a recent finding that low planting density appropriately promotes photosynthetic activity, increases the activity of lignin-related enzymes, and eventually enhances the lodging resistance of the strip intercropped soybean stems^39^. Other studies have shown that the activity of lignin-related enzymes (PAL, TAL, and CAD) play essential roles in plant lignin synthesis pathways^40,41^.

Crop yield is a critical factor in determining the quality of the planting methods. Crop yield is closely related to the accumulation of photosynthetic products^42^. When light is insufficient, crop shading occurs, resulting in slender crop stems, reduced dry matter accumulation, and reduced enzyme activity, making crops prone to lodging and affecting yield^43^. One of the essential approaches to increasing crop light energy utilization and light interception is to use an appropriate intercropping mode^44^. Moreover, intercropping can efficiently increase maize yield by producing complementary benefits between crops^45^. Expanding the row spacing between intercropped maize and soybean is more conducive to soybean growth^46^. In our study, compared to those under uniform-ridge pattern (U), the compound yields of maize and soybean under the narrow-wide row pattern (D) significantly increased by 5.97% and 5.78% in D-M6S6 and D-M4S6, respectively.

## Conclusions

Compared with the uniform-ridge pattern (65 cm), the narrow-wide-row planting pattern (80 cm and 50 cm) and intercropping ratio significantly enhanced the canopy photosynthetic characteristics, stem bending resistance and related physiological indices of soybean plants. In addition, the narrow-wide-row planting pattern remarkably improved the compound yield of the intercropping population.

With the continuous increase in mechanization, the traditional maize–soybean intercropping model cannot adapt to the whole mechanized process of modern crop production in China. Therefore, in actual production, only a combination of agricultural machinery and agronomy can achieve a high and stable yield and high efficiency. According to our results, the intercropping ratio with four rows of maize and six rows of soybean in a narrow-wide row planting pattern (D-M4S6) is more effective at improving the lodging resistance of soybean and weakening the negative impact of intercropping on soybean yield, effectively utilizing the complementary advantages of maize- soybean intercropping.

## Materials and Methods

### Ethics statement

No specific permission was required to conduct the field experiments. All experiments were performed according to the institutional guidelines of the Jilin Agricultural University of Changchun, China. In addition, all plant collections complied with the IUCN Policy Statement on Research Involving Species at Risk of Extinction and the Convention on the Trade in Endangered Species of Wild Fauna and Flora.

### Experimental site

Field trials were conducted in 2021 and 2022 at the Western Research Farm of the Faculty of Jilin Agricultural University (124° 48′ E longitude and 45° 08′ N latitude). Figure 9 shows the average monthly rainfall and temperature over the two years. The soil at the test site was a chernozem, with an organic matter content of 1.40% in the 0–20 cm soil layer, a total nitrogen content of 2.133 g kg^–1^, a total phosphorus content of 353.83 mg kg^–1^, and alkaline hydrolysis nitrogen, available phosphorus, and available potassium contents of 75.91 mg kg^–1^, 16.31 mg kg^–1^, and 130.24 mg kg^–1^, respectively, and a soil pH of 7.24.

### Experimental design

The tested maize hybrid, Tianyu 108 (126 days from emergence to maturity), was obtained from Yuntianhua Group Co., Ltd. in Changchun, Jilin Province, China. The soybean variety Jinong 40 (127 days from emergence to maturity) was the soybean variety obtained from the Agricultural College of Jilin Agricultural University. The test adopted a split-plot design, the main area factor was the planting pattern (narrow-wide row planting pattern and uniform-ridge planting pattern), and the subarea factor was the four intercropping ratios. In compliance with mechanized harvesting, eight treatments with three replicates of 13,000 m^2^ each plots were designed. The planting densities of maize and soybean were 70,000 plants ha^–1^ and 200,000 plants ha^–1^, respectively. Figure 10 shows the eight treatments, which included intercropping with four rows of maize and four rows of soybean in a uniform-ridge planting pattern (U-M4S4), four rows of maize and four rows of soybean in a narrow-wide row planting pattern (D-M4S4), four rows of maize and six rows of soybean in a uniform-ridge planting pattern (U-M4S6), four rows of maize and six rows of soybean in a narrow-wide row planting pattern (D-M4S6), six rows of maize and six rows of soybean in a uniform-ridge planting pattern (U-M6S6), six rows of maize and six rows of soybean in a narrow-wide row planting pattern (D-M6S6), soybean monoculture in a uniform-ridge planting pattern (U-S), and soybean monoculture in a narrow-wide row planting pattern (D-S). Figure 10 shows the treatment tested. A narrow-wide row planting pattern (D) was adopted with a narrow row spacing of 50 cm and a wide row spacing of 80 cm, and the row height was 30 cm. A uniform-ridge planting pattern (S) was adopted with an equal row spacing of 65 cm, and the height of the planting mound was 30 cm All treatments were repeated three times.

### Crop management

Maize and soybean seeds were planted simultaneously on 28 April 2021, and 29 April 2022. Maize was fertilized with 230 kg of N ha^–1^,120 kg of P_2_O_5_ ha^–1^_,_ and 160 kg of K_2_O ha^–1^. Only 70% of the total N was initially applied, with P_2_O_5_ and K_2_O used as base fertilizers. The remaining 30% of the N fertilizer was topdressed at the flowering stage. The amounts of P_2_O_5_ and K_2_O applied to the soybean crops were 60 and 25 kg ha^–1^, respectively. The total amount of fertilizer applied was the base fertilizer. Maize and soybeans were harvested simultaneously on September 30, 2021, and September 28, 2022.

### Equipment and methodology

**Figure 10:**
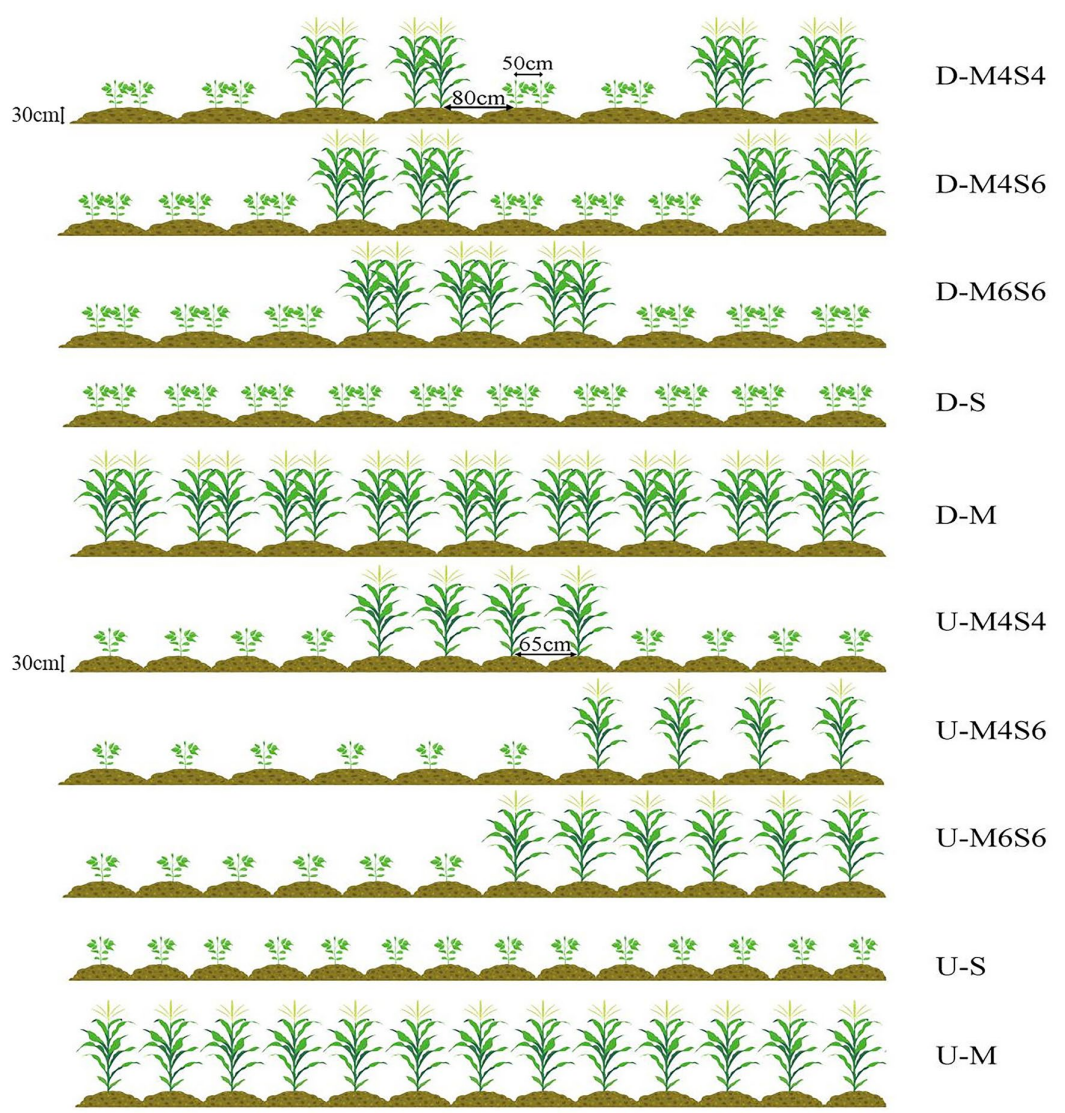
Diagrammatic sketches of the different planting patterns. (U: uniform-ridge planting pattern, with a row width of 65 cm and a row height of 30 cm; D: narrow-wide-row planting pattern with a wide row width of 80 cm, a narrow row of 50 cm, and a row height of 30 cm; M4S4: maize‒soybean 4:4 intercropping ratio; M4S6: maize‒soybean 4:6 intercropoping ratio; M6S6: maize‒soybean 6:6 intercropping ratio; S: soybean monoculture; M: maize monoculture) (the same applies to the all figures).

### Leaf area Index (LAI), photosynthetically active radiation (PAR), light transmittance ratio and stem bending resistance

Six soybean plants with consistent growth in each plot were randomly tested, and three replicates were performed, for a total of 18 plants evaluated per treatment. At 30, 45, 60, 75, and 90 days after sowing, the leaf area index (LAI) and photosynthetically active radiation (PAR) of the soybean canopy in each treatment were measured using a digital plant canopy imager (CI-110, Zealquest Scientific Technology Co., Ltd., China). During the initial flowering stage (R1), initial pod stage (R3), and initial grain stage (R5), we divided the soybean canopy into three parts, the bottom, middle, and upper leaves. The upper part represents the average points on the upper leaves of each row of soybeans; the middle part represents the average points on the middle leaves of each row of soybeans; and the bottom part represents the average points on the bottom leaves of each row of soybean. The light transmittance ratio of the soybean plants in each treatment was measured using a digital plant canopy imager. To assess stem bending resistance, soybeans plants were harvested from each treatment at the 3rd, 4th, and 5th nodes during the initial flowering stage (R1), initial pod stage (R3), and initial grain stage (R5). Stem bending resistance was measured using a Digital Force Tester (YYD-1, Zhejiang Top Instrument Hangzhou, China).

### Cellulose, Hemicellulose, and Lignin Contents of Soybean Stems

The cellulose content was determined according to the Seifert method using a mixture of acetylacetone, 1,4-dioxane, and concentrated hydrochloric acid^47^. The prepared mixture containing 1 g of lignocellulosic material was heated for 30 min in a water bath at 100 °C. The samples were filtered and washed successively with methanol, dioxane, hot water, and methanol. The components of the soybean stem fractions were analysed using Fourier transform infrared spectroscopy (FTIR) and X-ray diffraction (XRD). The cellulose content was calculated using the following formula^48^:

1 $$Cellulose\, content(mg\cdot {g}^{-1})=\frac{4.76(\Delta A+0.0043)}{W}$$

where ∆A = Light absorbance value W = Dry sample weight.

The hemicellulose content was determined using the Xiong SM method^49^. The hydrochloric acid hydrolysis method was used to evaluate the hemicellulose content, and the lignin content was determined using the Klason method^50^. After hydrolysis with 72% H_2_SO_4_, insoluble material in the stem cell walls was quantified using this method. A spectrophotometric technique was used to determine the amount of acid-dissolved lignin. The concentration of acid-soluble lignin in various stem samples following acid hydrolysis was determined using ultraviolet–visible spectroscopy. The lignin content was calculated using the following formula^48^:

2 $$Ligning \,content (mg\cdot {g}^{-1})=\frac{0.075 \times \left(\Delta A -0.0068\right)}{W \times T}$$

where ∆A = Light absorbance value. W = Dry sample weight. T = Dilution ratio.

### PAL, TAL and CAD Activities of in Soybean

The activities of phenylalanine ammonia-lyase (PAL) and tyrosine ammonia-lyase (TAL) activity were measured using Abell’s method^51^. The enzyme extract (0.5 mL) was mixed with 2.5 mL of a saturated solution of 12 mM phenylalanine or tyrosine in 0.1 M Tris/HCL buffer (pH 8.5) as substrate, respectively. PAL and TAL activities were measured at 290 and 315 nm, respectively, and are expressed as U/(g·FW·h).

The activity of cinnamyl alcohol dehydrogenase (CAD) was evaluated using the method described by Morrison^52^. The CAD reaction mixture (3 ml) comprised 1 mL of 1 mol L^–1^ trans-cinnamic acid and 1 mL of 0.5 mol L^–1^ phosphate buffer. The reaction mixture was mixed with crude enzyme extract and incubated for 30 min in a 37 °C water bath. Enzyme activity was measured spectrophotometrically by measuring the absorbance at 340 nm. Enzyme activity was expressed as U/(g·FW·h).

### Yield and land equivalent ratio index (LER)

When the maize and soybean plants were physiologically mature, ten sample plants were chosen to determine the number of ears and grains and the number of pods and effective grains per pod in soybean. The 100-grain weights of maize and soybean were recorded after natural air drying, and the water content was measured using a special water tester. The maize and soybean harvests in 2021 and 2022, respectively , were corrected by a 14% water content and converted to yield per unit area. According to the intercropping ratio of each treatment, the yield of soybean strips and the yield of maize strips were converted into the compound yield of soybean and the compound yield of maize, respectively. The combined production of the maize and soybean populations is known as the compound yield. The following formula was used to calculate the LER:

3 $${\text{LER}}=\frac{{\text{Yim}}}{{\text{Ymm}}}+\frac{{\text{Yis}}}{{\text{Yms}}}$$

where Yim is represents the maize yield under strip intercropping, Ymm is the maize yield under monoculture, Yis is the soybean yield under strip intercropping, and Yms is the soybean yield under monoculture.

### Statistical analysis

The data are presented as the mean ± standard errors. Two-way ANOVA was used to analyse the data using SPSS software (version 19.0). Each stage was examined separately, and Duncan’s test was used to evaluate differences between treatments (p = 0.05). Amos 24.0 was used to construct a structural equation to reveal the effects of planting patterns and intercropping ratios on the stem bending resistance and yield of soybean, and the comparative fitting index (CFI), goodness-of-fit index (GFI) and root mean square error of approximation (RMSEA) were used to evaluate the goodness of fit of the structural equation model.

## Data Availability

The datasets used and/or analysed in this study are available from the corresponding author upon reasonable request.
